# Assessing the Impact of AI Education on Hispanic Healthcare Professionals’ Perceptions and Knowledge

**DOI:** 10.3390/educsci14040339

**Published:** 2024-03-22

**Authors:** Frances Heredia-Negrón, Eduardo L. Tosado-Rodríguez, Joshua Meléndez-Berrios, Brenda Nieves, Claudia P. Amaya-Ardila, Abiel Roche-Lima

**Affiliations:** 1 CCRHD RCMI-Program, Medical Sciences Campus, University of Puerto Rico, San Juan, PR 00934, USA;; 2 Department of Biostatistics and Epidemiology, Medical Science Campus, University of Puerto Rico, San Juan, PR 00934, USA;

**Keywords:** AI, medical school, Hispanics, healthcare

## Abstract

This study investigates the awareness and perceptions of artificial intelligence (AI) among Hispanic healthcare-related professionals, focusing on integrating AI in healthcare. The study participants were recruited from an asynchronous course offered twice within a year at the University of Puerto Rico Medical Science Campus, titled “Artificial Intelligence and Machine Learning Applied to Health Disparities Research”, which aimed to bridge the gaps in AI knowledge among participants. The participants were divided into Experimental (*n* = 32; data-illiterate) and Control (*n* = 18; data-literate) groups, and pre-test and post-test surveys were administered to assess knowledge and attitudes toward AI. Descriptive statistics, power analysis, and the Mann–Whitney U test were employed to determine the influence of the course on participants’ comprehension and perspectives regarding AI. Results indicate significant improvements in knowledge and attitudes among participants, emphasizing the effectiveness of the course in enhancing understanding and fostering positive attitudes toward AI. Findings also reveal limited practical exposure to AI applications, highlighting the need for improved integration into education. This research highlights the significance of educating healthcare professionals about AI to enable its advantageous incorporation into healthcare procedures. The study provides valuable perspectives from a broad spectrum of healthcare workers, serving as a basis for future investigations and educational endeavors aimed at AI implementation in healthcare.

## 1. Introduction

Artificial intelligence (AI) has experienced remarkable advancements in recent years, driven by the synergy of machine learning techniques and the availability of vast amounts of data [1]. In parallel, AI has permeated various aspects of our daily lives, exemplified by the proliferation of virtual assistants like Chat-GPT, which engage in natural language conversations [2,3]. These developments underscore the profound influence of AI on society, with the potential to revolutionize numerous domains, none more significant than healthcare.

The healthcare sector benefits immensely from integrating AI, promising transformative changes in patient care, diagnosis, and treatment [4–6]. Among the most prominent AI applications in healthcare is the analysis of radiological images. AI algorithms have achieved impressive success detecting anomalies and assisting medical professionals in making critical decisions [7–9]. Furthermore, AI has demonstrated its prowess in addressing specific healthcare challenges, including breast cancer detection [10] and the rapid diagnostics of diseases like COVID-19 [11]. These achievements represent only a fraction of AI’s potential to revolutionize healthcare, and their implications extend far beyond medical imaging.

However, alongside these remarkable advancements come significant controversies and concerns surrounding the integration of AI in healthcare. Among these concerns is the apprehension that AI might replace human doctors and disrupt the doctor-patient relationship [12,13]. Furthermore, there is a growing debate regarding whether healthcare professionals should rely on AI systems for clinical decisions or exercise their judgment [14]. Amid these controversies, there are compelling arguments supporting the potential benefits of AI in healthcare, including improved diagnostic accuracy, efficient data analysis, and the augmentation of medical expertise [15].

Existing studies assessing training of AI in healthcare professionals have found that while healthcare professionals acknowledge the potential benefits of AI, there is a significant lack of preparation regarding concepts and applications [13,16,17]. The majority are not aware of AI’s role in their daily work and cannot distinguish between different AI technologies such as machine learning and deep learning. Despite these gaps in knowledge, there is optimism about AI’s utility in the medical field. These findings highlight the necessity for improved AI education among healthcare workers to foster its beneficial integration into healthcare practices.

In health-related data science, diversifying teams is a key strategy to tackle or prevent health disparities. Diverse teams, bringing varied perspectives, ideas, and feelings, are better positioned to devise innovative and thorough solutions that take into account different viewpoints. This not only fosters creativity but also enhances problem-solving, widens the talent pool, and is crucial for sustained economic growth. Despite this, minorities are underrepresented in data science research, employment, and the data itself. For example, Hispanics make up 18% of the U.S. population but the data available for this group are insufficient. Moreover, they account for just 8% of students enrolled in data science or related programs in the U.S., compared to 50% being Caucasian [18]. Education and motivation of biomedical professionals from diverse backgrounds are vital.

The primary objective of this study is to investigate the awareness and perceptions of AI among Hispanic healthcare-related professionals, recognizing the importance of understanding their viewpoints in the context of AI integration in healthcare. This study aligns with the broader research effort to assess AI awareness and perceptions among healthcare professionals [19]. Notably, this study also stands out as the first of its kind within a Hispanic institution, offering unique insights into the perspectives of a diverse healthcare workforce within this specific demographic context.

## 2. Materials and Methods

### 2.1. Course Description and Participant Selection

We have previously implemented the course “Artificial Intelligence and Machine Learning Applied to Health Disparities Research (AIML + HDR)” to address the biases in AI and Machine Learning (ML) in minority health disparities. This course was developed as part of the funded “Notice of Special Interest (NOSI): Administrative Supplements to Enhance Data Science Capacity at National Institute of Minority and Health Disparities, National Institutes of Health”, at the University of Puerto Rico, Medical Science Campus. AIML + HDR course aimed to leverage data science (DS) principles in the field of health disparities, particularly focusing on the Hispanic population. The curriculum included an introduction to the Jupyter Notebook Framework, programming in R and Python for data manipulation, and the utilization of ML libraries to construct predictive models. It also delved into critical issues like social determinants of health and the inherent bias in data. More comprehensive details and outcomes of the course were discussed in the original publication Heredia et al., 2023 [18]. The course was provided twice, the first edition from April to June 2022, and the second edition from February to April 2023.

Course registration forms were opened for students and staff from the University of Puerto Rico and other minority health research/educational institutions interested in taking the course. Out of the pool of registered people, course participants were selected based on several criteria defined in the project. These criteria included clinicians, health practitioners, as well as investigators, graduate/undergraduate students, and technical staff related to health, prioritizing health disparities research or related fields. Summing up both course editions, a total of 50 participants finished the course and completed the pre-test and post-test surveys. All the participants were adults (21 women and 29 men), and 45 were self-reported Hispanics. Their professional backgrounds were health-related academic researchers (11 participants); health-related students (26 participants); medical staff (9); and health-related data scientists (4 participants). For the analysis in this paper, the 50 participants were divided into two groups, based on their initial data science knowledge: the “Experimental Group” which included data-illiterate participants (32) and a “Control Group” that included data-literate participants (18), as described in Table 1. This division was defined based on the information the participants provided when they completed the registration forms.

### 2.2. Surveys

Two different tests were completed by the participants: a pre-test before starting the course, and a post-test after the course completion. Questions of these tests are described in Table 2. These tests were based on the survey introduced by Castagno and Khalifa in 2020 [20], which assesses the awareness of AI technologies among healthcare professionals and explores their perceptions of AI applications in medicine. The data obtained from the pre- and post-tests have been used for the analysis in this paper. Questions were grouped into two sections, as follows: Knowledge base (Questions 1–4) to examine the participants’ familiarity with AI, and Attitudes (Questions 5–9) to inquire into participants’ viewpoints and concerns regarding AI.

The structure of our questionnaire aligns with the CHERRIES (Checklist for Reporting Results of Internet E-Surveys) guidelines [21], ensuring clarity, categorization, and qualitative insights into the perceptions and knowledge of healthcare-related participants concerning AI. The decision to employ the term “useful” without further elaboration in questions Q7 and Q9 was motivated to capture both present and future perceptions of AI applications, a methodology consistent with previous research in this domain [22].

### 2.3. Statistical Analysis

Descriptive statistics provided an overview of the data for each question. This involved calculating each test group’s mean, median, standard deviation, and distribution quartiles. To assess the statistical significance of differences between the two groups (Experimental versus Control), we employed the Mann–Whitney non-parametric statistical test. The Mann–Whitney test determined whether there were significant differences between (95%) by groups for each question. For the analyses, we used the program GraphPad PRISM version 10.0.3.

In addition, statistical power for the survey’s total score was evaluated using the Wilcoxon signed-rank test. The methodology involved specifying the effect sizes observed, adhering to a significance level of alpha = 0.05, and documenting the sample sizes for the analytical groups, with the experimental group consisting of 32 participants and the control group comprising 18 participants. Following these steps, the achieved power (1—beta) was computed to determine the study’s ability to detect true effects. This analysis was conducted using Stata 16.1 software and G*Power version 3.1.9.7, selected for its capability to meet the specific demands of the Wilcoxon signed-rank test, ensuring a robust and accurate power analysis tailored to the study’s requirements.

## 3. Results

### 3.1. Descriptive Statistics

**Figure 1.**
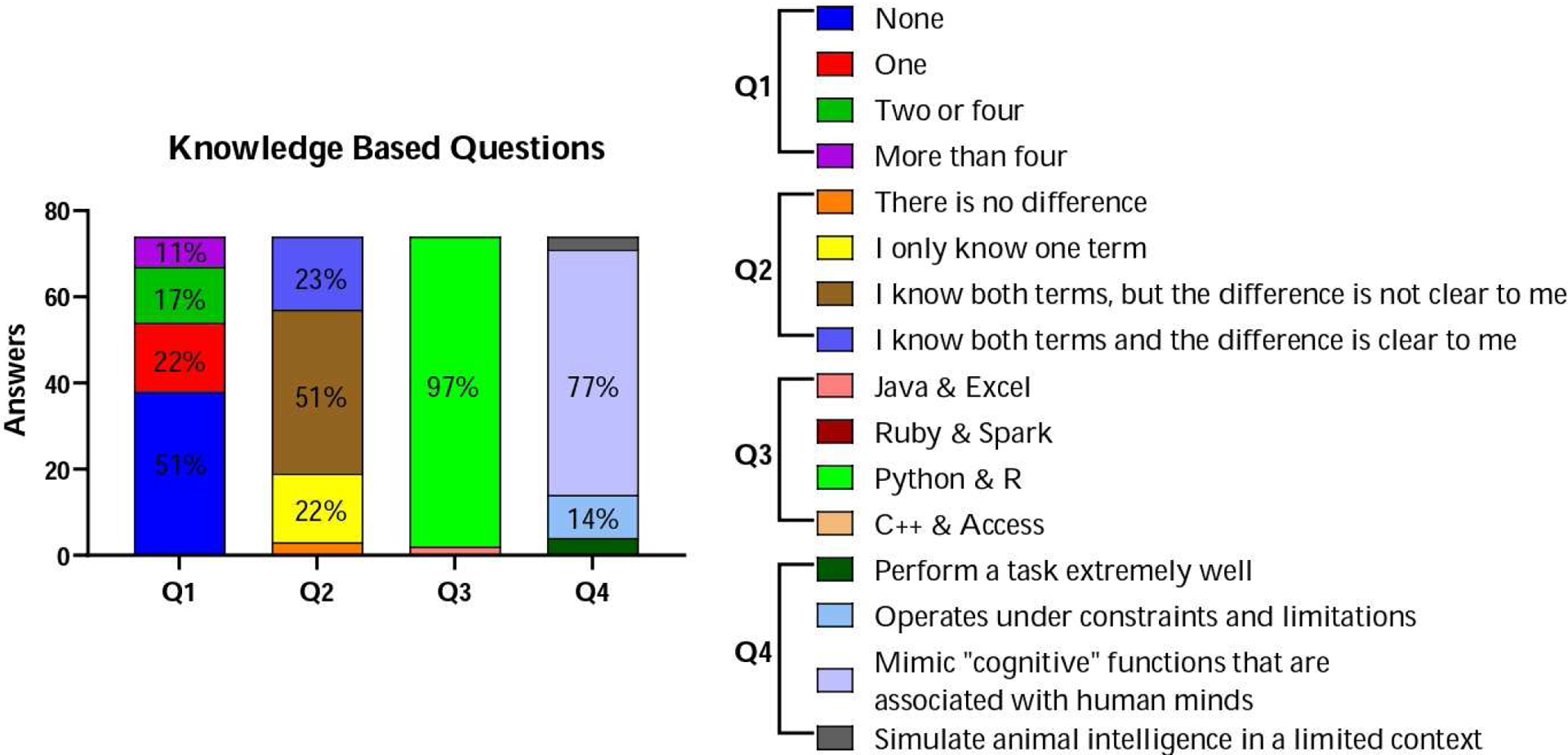
Participants’ knowledge base regarding artificial intelligence (AI) and its applications. The average of each response per category is shown. Areas without numbers have fewer than 10.0% of answers.

These results were obtained by analyzing the pre-test survey data for each question. In relation to the Knowledge base section, the results can be found in Figure 1 (Q1–Q4). Question 1, which inquired about the number of AI applications encountered in their work, shows a proportion of 51% of the participants having encountered none; meanwhile, 22% had come across a single AI application, 18% had encountered between two and four, and a more minor yet notable 9% had experience with more than four AI applications (Figure 1—Q1). In Question 2, participants were asked about their understanding of the distinctions among artificial intelligence, machine learning, and deep learning, a minority of 4%, believed there was no difference between these terms, while 22% indicated awareness of just one term; however, the majority, comprising 51%, admitted to knowing both terms but found the differences unclear, indicating a need for further clarification; and another 22% expressed clarity in distinguishing these concepts (Figure 1—Q2). Question 3 inquired about identifying two critical programs in data science, where Python and R emerged as clear front-runners with a staggering 97% consensus; the limited endorsement of positive answers for the other options, such as Java and Excel (3%) and Ruby and Spark (0%), underscored the dominant presence of Python and R as the answer (Figure 1—Q3). Finally, Question 4 investigated how participants perceived the idea of artificial intelligence. Most respondents, 77%, believed that AI mimics “cognitive” functions found in human minds, underscoring the relevance of AI’s capacity to emulate human mental processes, while 14% acknowledged that AI was subject to restrictions and limitations, 5% thought AI’s primary function was to perform tasks extremely well, and only 4% of respondents connected AI to the constrained modeling of animal intelligence (Figure 1—Q4).

**Figure 2.**
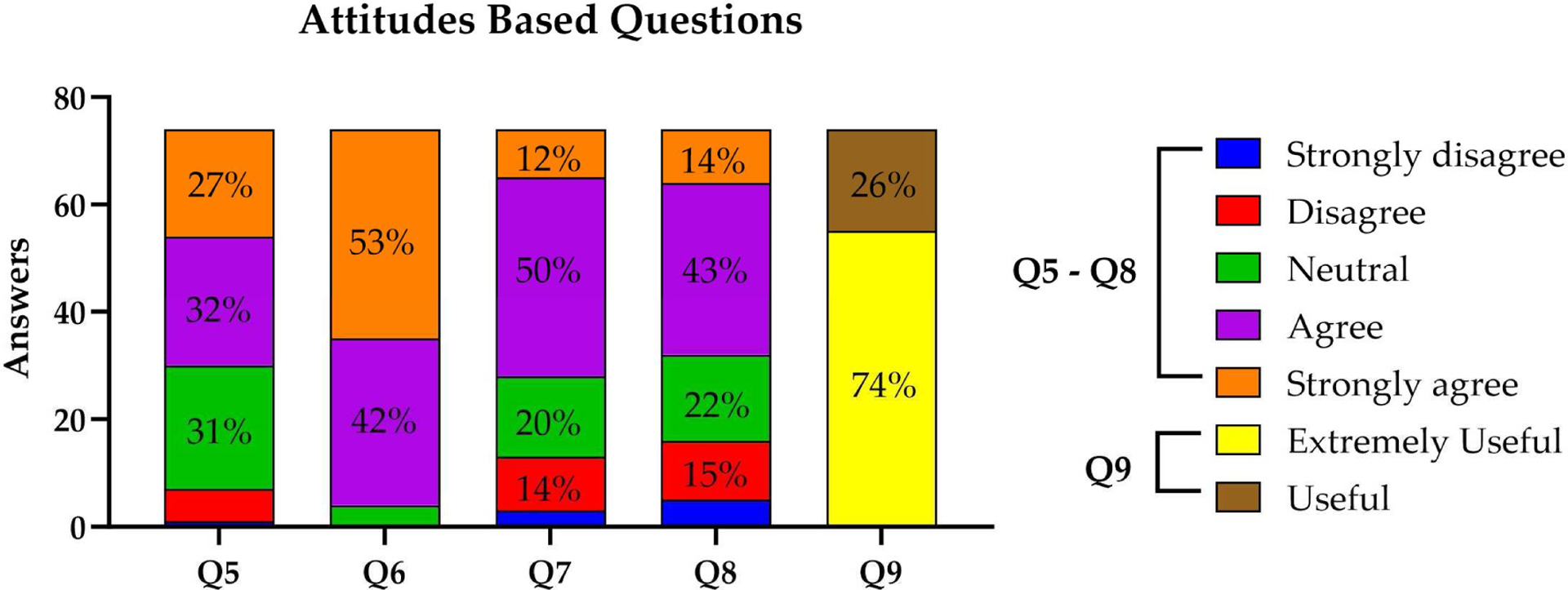
Participants’ Attitudes and worries regarding artificial intelligence (AI) and its applications. The average of each response per category is shown. Areas without numbers have fewer than 10.0% of answers.

Concerning participants’ Attitudes type of questions, the results can be seen in Figure 2 (Q5–Q9). Question 5 explored the consideration of ethical issues, where a significant 59% of participants strongly agreed or agreed that there may be severe ethical concerns; on the other hand, 8% disagreed, and 1% strongly disagreed, representing the minority with less concern regarding the ethical implications; while a substantial portion, 31%, remained neutral, indicating a potential ambivalence or a need for further exploration of the topic (Figure 2—Q5). In Question 6, a substantial 53% of respondents strongly agreed with the utility of these AI skills, while 42% agreed, signifying a collective consensus on the practicality of these competencies in everyday life; and a minimal 5% reported neutrality (Figure 2—Q6). The responses regarding Question 7 were varied; while 62% of participants strongly agreed or agreed that they comprehended these applications, 18% disagreed or strongly disagreed, and 20% remained neutral reflecting a segment with limited understanding or skepticism (Figure 2—Q7). Similarly, in Question 8, the majority, 43%, agreed, with an additional 14% strongly agreeing, indicating that a significant proportion acknowledged the connection between AI and health disparities; and conversely, 22% disagreed or strongly disagreed, meanwhile a notable 22% remained neutral (Figure 2—Q8. Finally, Question 9, which examined the perceived utility of AI in participants’ respective areas of work, obtained a majority, 74%, who regarded AI as extremely useful, while 26% found it helpful (Figure 2—Q9).

### 3.2. Comparison of Experimental versus Control Groups

**Figure 3.**
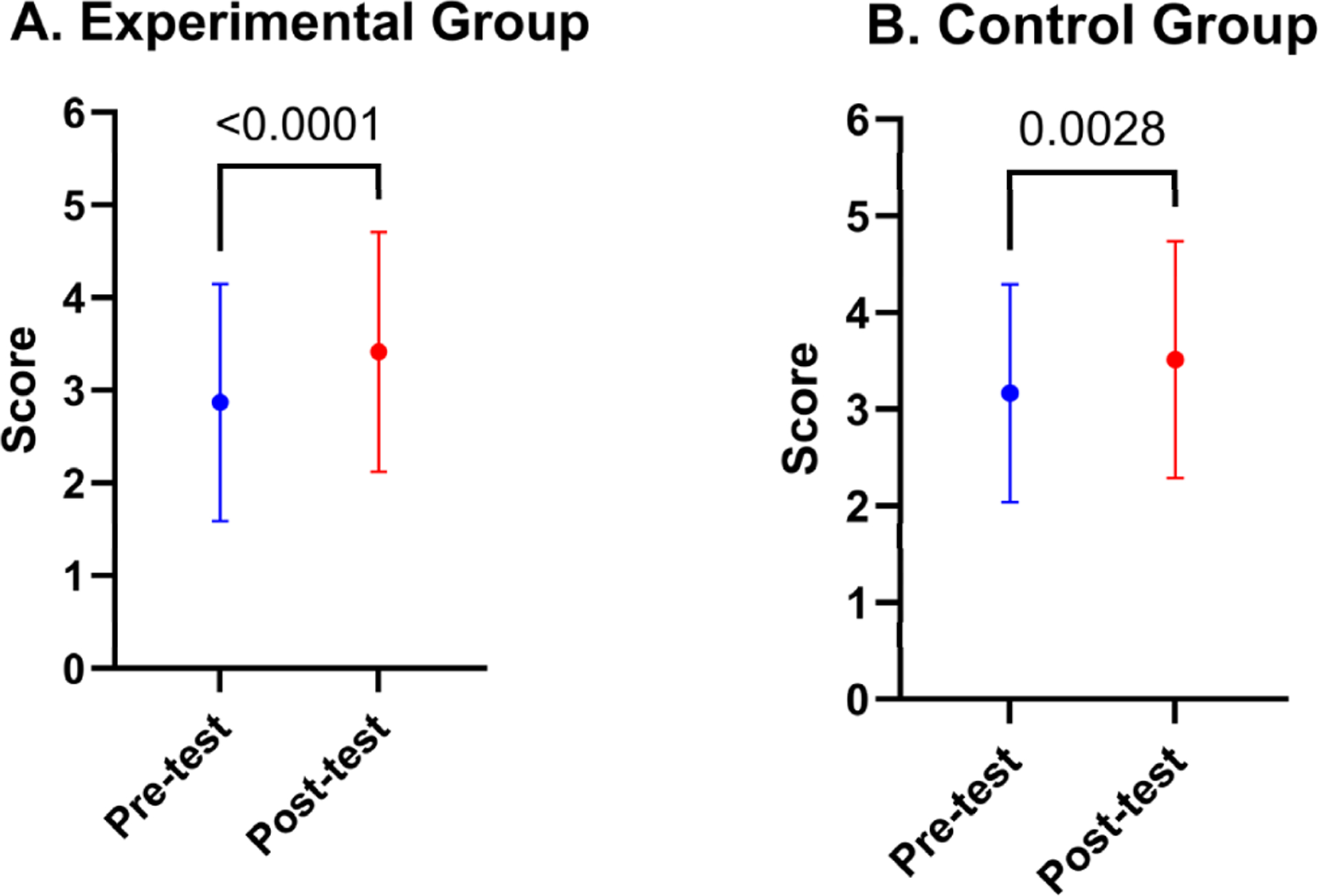
Comparing all questions together pre-test and post-test scores means with ± standard deviation among our participant groups. (**A**) Comparison for the Experimental Group (data-literate). (**B**) Comparison for the Control Group (data-literate). A *p*-value of less than 0.05 was considered statistically significant.

**Figure 4.**
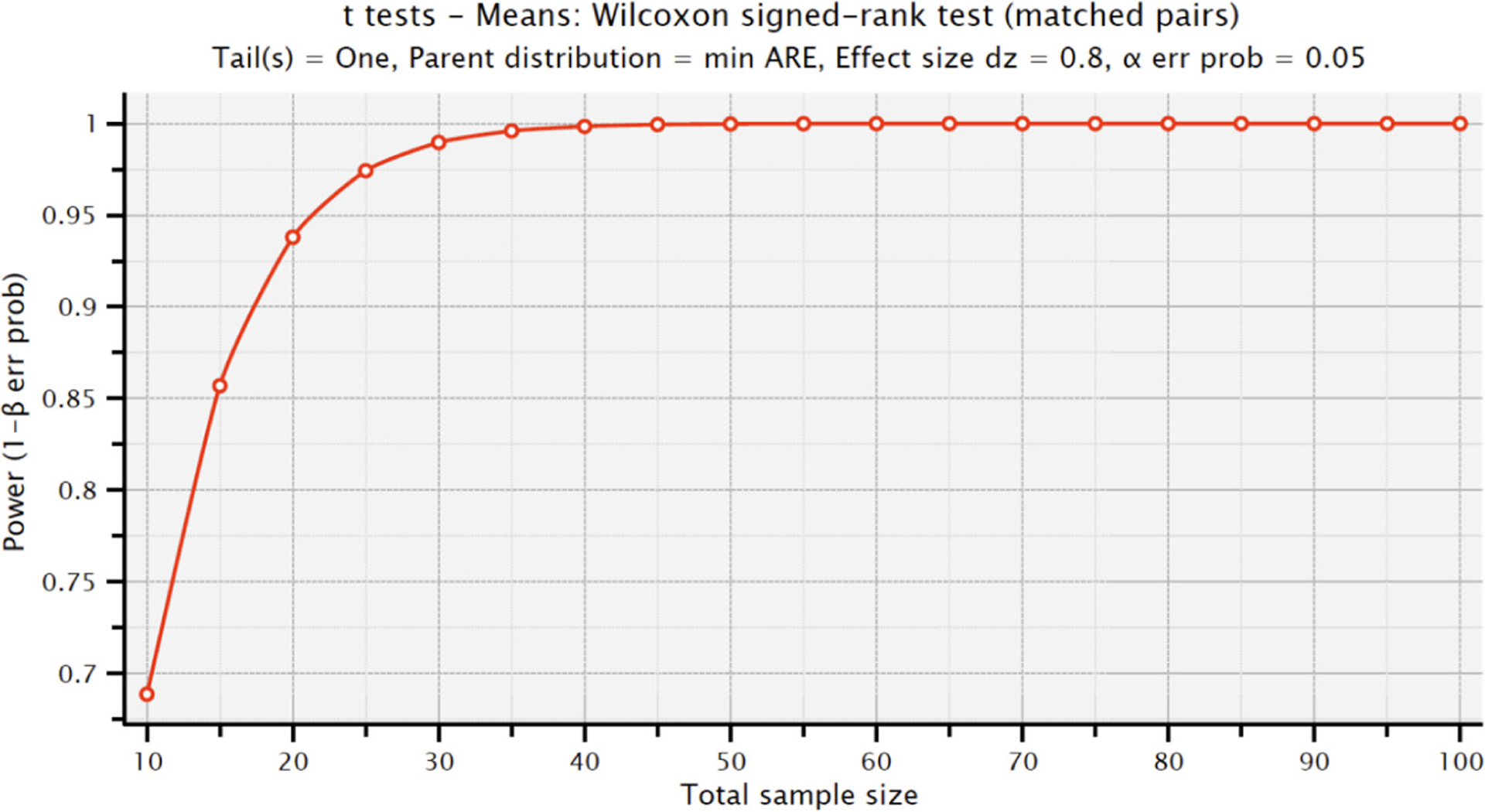
Statistical power for the survey’s total score in the experimental group.

Firstly, we compared the results of averaged all-together question values, which can be found in Figures 3 and 4. On average, the Experimental Group (data-illiterate) participants showed a considerable improvement, going from a pre-test mean score of 2.872 to a post-test mean score of 3.417, with a highly significant *p*-value of less than 0.0001 (Figure 3A) (Table S1). With a less noticeable increase from a pre-test mean score of 3.167 to a post-test mean score of 3.512, the Control Group (data-literate participants) also showed some progress, yielding a statistically significant *p*-value of 0.0028 (Figure 3B) (Table S1). Figure 4 shows the statistical power of the survey’s total score. As can be seen, for our experimental group size of 32 participants, a power value of greater than 0.98 is obtained with the observed effect size of 0.8 and a significance level of alpha = 0.05.

**Figure 5.**
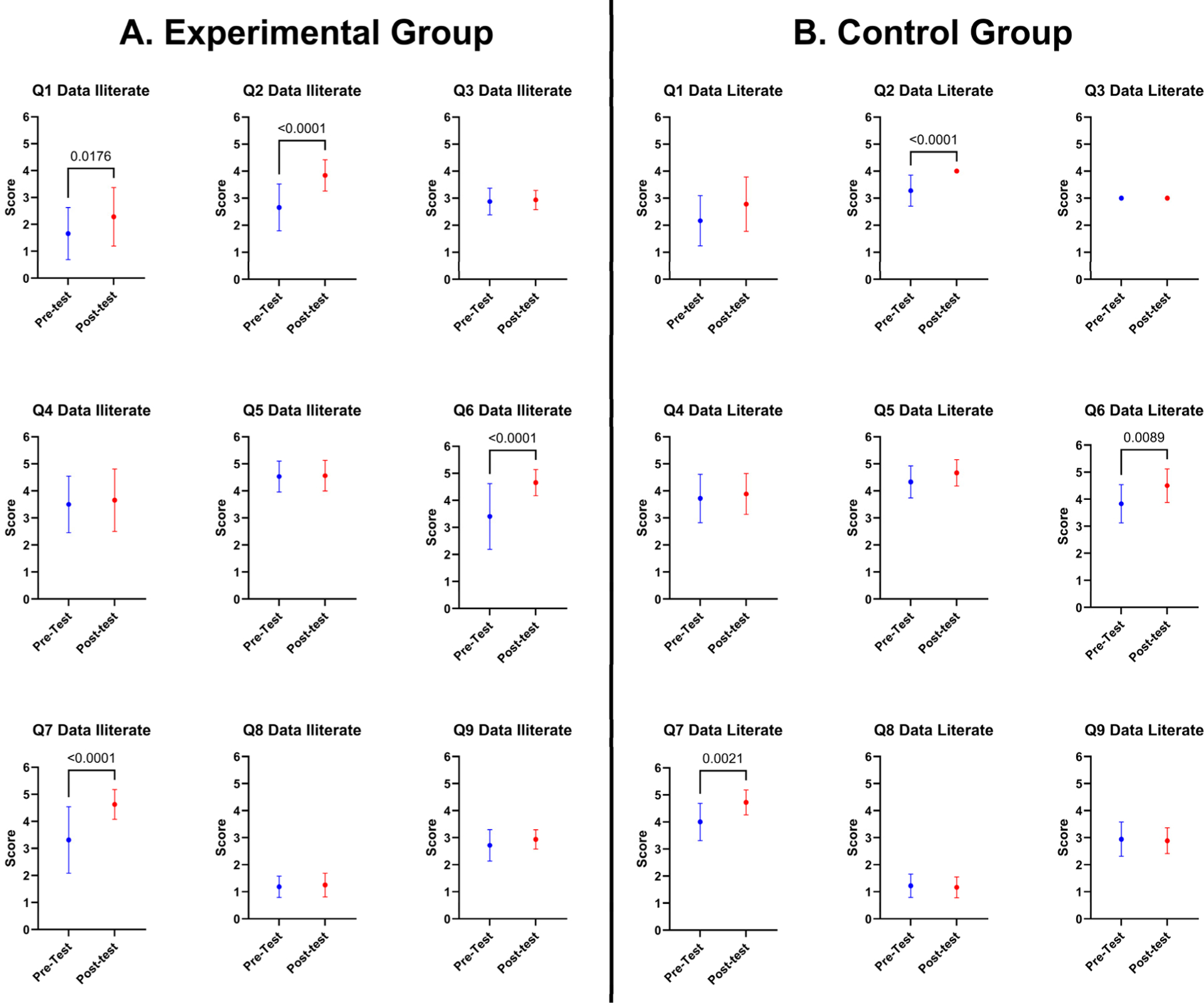
Comparing the pre-test and post-test scores means with ± standard deviation per question among for each group. (**A**) Comparison for the Experimental Group (data-literate). (**B**) Comparison for the Control Group (data-literate). A *p*-value of less than 0.05 was considered statistically significant.

Secondly, we compared the groups by questions (Figure 5). For the Experimental Group (Figure 5A), significant gains were noted in Knowledge based questions, Question 1 and Question 2. Question 1 had a statistical *p*-value of 0.0176, demonstrating a significant improvement in comprehension (Figure 5A—Q1). Similarly, Question 2 showed significant improvement, with a remarkably low *p*-value of <0.0001 (Figure 5A—Q2). Regarding questions based on Attitudes, notable improvements were observed in Question 6 and Question 7. The pre-test means scores for Q6 and Q7 grew from 3.406 to 4.656 and 3.313 to 4.625, respectively, and both produced highly significant *p*-values of less than 0.0001 (Figure 5A—Q6, Figure 5A—Q7) (Table S1).

In the statistical analysis of the Control Group (data-literate participants), significant improvements were identified only for three questions. For the Knowledge based domain, only Question 2 exhibited a notable increase from a pre-test mean score of 3.278 to a post-test mean score of 4, with a *p*-value < 0.0001, signifying a substantial enhancement in their understanding (Figure 5B—Q2). For Attituded domain questions, significant positive shifts were observed only in Question 6 and Question 7, with an associated *p*-value of 0.0089 and 0.0021, respectively (Figure 5B—Q67; Figure 5B—Q7).

## 4. Discussion

In this study, we aimed to analyze the awareness and perceptions of artificial intelligence applied to health disparities among participants related to health. Knowledge base and Attitude assessments, conducted through a nine-question survey, revealed an overall positive attitude towards artificial intelligence among the participants.

Regarding Knowledge based questions, most indicate that AI would be extremely useful in their work area, as shown also by Mahajan et al., 2019, and Yang et al., 2022 [23,24]. Comparing our results on the practical exposure of students to AI applications with the existing literature, our findings align with the current state of AI adoption in other health-related fields. A study of the perception of AI from medical and dental students [17] reported that 20.4% were not at all familiar with artificial intelligence and robotics in medicine, and 40.1% were slightly familiar. In another study about the attitudes of United Kingdom (UK) [16] health-related participants regarding artificial intelligence, only 9.2% of students surveyed had received some form of teaching on AI. A substantial percentage of health professionals reporting no encounters with AI applications emphasizes the ongoing challenge of effectively incorporating AI tools into educational and professional environments. This highlights a gap that educational institutions need to address to enhance practical exposure and prepare students for the increasing role of AI in their respective fields. On assessing our distinctions between artificial intelligence, machine learning, and deep learning, our participants expressed having heard of the terms but needed clarification. Similarly, in a study of UK medical students’ attitudes towards AI [16], nearly half the respondents reported feeling uncomfortable with the nomenclature associated with AI (disagree and strongly disagree, 43.4%); in comparison, only 30.2% reported that they did. The study of the perception of AI among medical and dental students [17] indicated that the majority of the students said they lack an understanding of the technologies that underpin “artificial intelligence” and “deep learning”. A third study about US medical students’ perceptions of artificial intelligence [25] found that most students (86.1%) lacked knowledge regarding core AI concepts. These results underscore the necessity for further education in these fundamental AI concepts. The existence of a substantial segment (22%) in our cohort with a clear understanding is encouraging, yet addressing the prevalent uncertainty is crucial for building a foundation for stacking more skills in the future. The participants’ perceptions of AI, particularly the belief that AI mimics “cognitive” functions found in human minds (77%), resonates with previous research, indicating a widespread acknowledgment of AI’s capacity for cognitive simulation. For example, a study that explored medical students’ and faculty’s perceptions of artificial intelligence [26] found that only 8.7% of the survey respondents disagreed: “The diagnostic ability of artificial intelligence is superior to the diagnostic ability of a human doctor”. A conceptual analysis article [26] that aims to set a framework for understanding AI intelligence stated, “Our aspiration of constructing AGI systems possessing humanoid intelligence is the premise that human (general) intelligence is the ‘real’ form of intelligence.” This paper shows that while there is a consensus on AI’s cognitive capabilities, there remains room for exploring and expanding participants’ awareness of AI’s multifaceted roles and potential applications beyond the cognitive emulation of humans.

In relation to Attitude related questions, our participants indicated harboring reservations about the ethical implications of AI. This finding contrasts with the finding of a similar study exploring how medical students from Germany, Austria, and Switzerland perceive the use of AI in medicine [27]. Despite their varied prior exposures, 71.7% anticipated a positive impact of AI on medicine [27]. The disagreement suggests a divide in perspectives, emphasizing the importance of fostering a nuanced understanding of AI’s ethical challenges. The consensus of 95% of our participants toward the practical benefits of AI aligns with the growing integration of AI and data science into various aspects of modern life. The varied responses regarding comprehension of AI applications in medicine reveal a spectrum of awareness within our participants. A very similar result was found in a study about medical students’ attitudes toward AI by Kimmerle et al., 2023 [28]. In this study, 58.62% of the respondents indicated that they had some level of understanding of AI applications in medicine. The neutral responses (20%) suggest an opportunity for enhanced education and communication regarding AI’s role in the medical field. Our findings regarding participants’ understanding of AI’s role in addressing health disparities reflect a mixed landscape. It indicates a need for targeting efforts to elucidate the potential of AI in mitigating health disparities, fostering a more informed perspective among health-related professionals working and researching on this topic. The overwhelming consensus on the perceived utility of AI in participants’ respective areas of work emphasizes a positive outlook on AI’s potential to bring value to professional domains. The positive perception of the effect of AI in the health care profession was also shared by the respondent of a survey on knowledge and attitudes of registered nurses about artificial intelligence [29]. In this survey, 70% agreed or strongly agreed AI would revolutionize health care. This result aligns with the broader trend of AI adoption in various industries. The positive perception suggests a readiness among participants to embrace AI technologies as valuable tools in their work [29].

Regarding the comparison within the Experimental Group, analysis of the significant gains was observed in our Experimental group, highlighting the course’s ability to bridge knowledge gaps. The remarkable improvement (*p*-vale < 0.0001) underscores the course’s effectiveness in enhancing comprehension and knowledge acquisition among students with limited initial data proficiency. Further analysis of Knowledge based questions (Q1 and Q2) revealed statistically significant progress, emphasizing the positive impact on fundamental understanding. Notable improvements in Attitudes, as indicated by increased pre-test mean scores in Question 6 and Question 7, further suggest a positive shift in perception among data-illiterate participants.

While Control Group participants showed a less pronounced increase in mean scores, the progress remains statistically significant (*p*-value = 0.0441). Enhancements were observed in Knowledge based just in Question 2. Additionally, Attitudes toward machine learning and data science, measured by Question 6 and Question 7, demonstrated a substantial positive shift. These findings indicate that the course reinforced existing knowledge and positively influenced attitudes, emphasizing the course’s value even for students with prior data proficiency.

Despite the valuable insights gained from this study, certain limitations should be acknowledged. The primary pool mainly consisted of participants from a specific institution, the University of Puerto Rico, Medical Science Campus, which potentially introduced biases associated with regional education environments and curricula. Future research could explore more extensive and diverse participant samples to address these limitations and build on the current study’s foundation. Including participants from various academic backgrounds and institutions would enhance the external validity of the findings. Longitudinal studies tracking participants’ knowledge retention and evolving attitudes over an extended period could provide deeper insights into the long-term impact of AI education.

## 5. Conclusions

The study examined how artificial intelligence, machine learning, and data science courses affected participants’ knowledge and attitudes toward AI. All the students improved, even those who knew much about data before the course. This suggests that even students with prior data expertise benefited substantially from the course, gaining a deeper understanding of specific AI concepts. The study also suggests that health-related professionals need to have training for AI in different levels of knowledge. Covering basic ideas and giving hands-on experience with AI can make health-related professionals better prepared for AI in healthcare.

The substantial gains in comprehension and attitudes, especially among data-illiterate participants, highlight the course’s effectiveness in catering to diverse participants backgrounds and fostering a more robust understanding of AI concepts and applications. By exploring the awareness and perceptions of AI among healthcare professionals, this research seeks to contribute valuable insights to the ongoing discourse surrounding the role of AI in healthcare, specifically in health disparities, addressing the concerns and expectations of those directly involved in patient care within a distinct cultural setting.

## Supplementary Material

Supplementary Heredia

## Figures and Tables

**Table 1.** Data knowledge of survey participants.

Data Knowledge	Number of Participants
Experimental Group (Data-illiterate)	32
Control Group (Data-literate)	18
Total	50

**Table 2.** The survey was divided into two sections: Knowledge base and Attitudes.

Sections	Questions		Answers
Knowledge base	(Q1) How many applications of AI have you come across in your work?	1.	None
		2.	One
		3.	Two to Four
		4.	More than four
	(Q2) Do you know the difference between artificial intelligence, machine learning and deep learning?	1.	There is no difference.
		2.	I only know one term.
		3.	I know both terms, but the difference is not clear to me.
		4.	I know both terms and the difference are clear to me
	(Q3) Which of the following are two of the most important programs in Data Science?	1.	Java & Excel
		2.	Ruby & Spark
		3.	Python & R
		4.	C++ & Access
	(Q4) The concept of artificial intelligence includes:	1.	Perform a task extremely well.
		2.	Operates under constraints and limitations.
		3.	Mimic “cognitive” functions that are associated with human minds.
		4.	Simulate animal intelligence in a limited context
Attitudes	(Q5) Do you think there may be serious ethical issues with the use of AI?	1.	Strongly disagree
		2.	Disagree
		3.	Neutral
		4.	Agree
		5.	Strongly agree
	(Q6) Reasoning skills used to understand Artificial Intelligence/Data Science can be helpful to my everyday life.	1.	Strongly disagree
		2.	Disagree
		3.	Neutral
		4.	Agree
		5.	Strongly agree
	(Q7) I understand how the topic of Artificial intelligence and machine learning are applied in medicine:	1.	Strongly disagree
		2.	Disagree
		3.	Neutral
		4.	Agree
		5.	Strongly agree
	(Q8) I understand how the topic of Artificial intelligence and Machine Learning applies to Health Disparities:	1.	Strongly disagree
		2.	Disagree
		3.	Neutral
		4.	Agree
		5.	Strongly agree
	(Q9) How useful do you think AI could be in your area of work?	1.	Extremely Useful
		2.	Useful
		3.	Of limited use
		4.	No use at all

## Data Availability

All data generated from this research are presented in this manuscript.
