# Supplementary material for: Assessing the Impact of AI Education on Hispanic Healthcare Professionals’ Perceptions and Knowledge
Source: Educ Sci (Basel). Author manuscript; Available in PMC 2024 May 30. (PMC11138866; doi:10.3390/educsci14040339)
Supplement: Supplementary Heredia [file NIHMS1993189-supplement-Supplementary_Heredia.pdf]

Supplementary Table S1. Descriptive statistics evaluating the pre-test and post-test scores considering the overall course questionnaire and each individual question for each participant group.

Question	Statistics	Experimental Group (n=32)		Control Group (n=18)	
		Pre-test	Post-test	Pre-test	Post-test
**Overall Course Questionnaire**	Minimum	1	1	1	1
	25% Percentile	2	3	3	3
	Median	3	4	3	4
	75% Percentile	4	4	4	4
	Maximum	5	5	5	5
	Mean	2.872	3.417	3.167	3.512
	Std. Deviation	1.277	1.293	1.127	1.227
	Std. Error of Mean	0.07527	0.0762	0.08855	0.09642
	Mann Whitney test	**P value: <0.0001**		**P value: 0.0028**	
**Q1**	Minimum	1	1	1	1
	25% Percentile	1	1	1	2
	Median	1	2.5	2	3
	75% Percentile	2	3	3	3.25
	Maximum	4	4	4	4
	Mean	1.656	2.281	2.167	2.778
	Std. Deviation	0.9708	1.085	0.9235	1.003
	Std. Error of Mean	0.1716	0.1917	0.2177	0.2365
	Mann Whitney test	**P value: 0.0176**		P value: 0.0544	
**Q2**	Minimum	1	1	2	4
	25% Percentile	2	4	3	4
	Median	3	4	3	4
	75% Percentile	3	4	4	4
	Maximum	4	4	4	4
	Mean	2.656	3.844	3.278	4
	Std. Deviation	0.8654	0.5741	0.5745	0
	Std. Error of Mean	0.153	0.1015	0.1354	0
	Mann Whitney test	**P value: <0.0001**		**P value: <0.0001**	
**Q3**	Minimum	1	1	3	3
	25% Percentile	3	3	3	3
	Median	3	3	3	3
	75% Percentile	3	3	3	3
	Maximum	3	3	3	3
	Mean	2.875	2.938	3	3
	Std. Deviation	0.4919	0.3536	0	0
	Std. Error of Mean	0.08695	0.0625	0	0

	Mann Whitney test	P value: 0.9999		P value: >0.9999	
Q4	Minimum	1	1	2	3
	25% Percentile	3	3	3	3
	Median	3.5	4	4	4
	75% Percentile	4	4.75	4.25	4.25
	Maximum	5	5	5	5
	Mean	3.5	3.656	3.722	3.889
	Std. Deviation	1.047	1.153	0.8948	0.7584
	Std. Error of Mean	0.1851	0.2038	0.2109	0.1788
	Mann Whitney test	P value: 0.4399		P value: 0.6146	
Q5	Minimum	3	3	3	4
	25% Percentile	4	4	4	4
	Median	5	5	4	5
	75% Percentile	5	5	5	5
	Maximum	5	5	5	5
	Mean	4.531	4.563	4.333	4.667
	Std. Deviation	0.5671	0.5644	0.5941	0.4851
	Std. Error of Mean	0.1002	0.09977	0.14	0.1143
	Mann Whitney test	P value: 0.9140		P value: 0.1326	
Q6	Minimum	1	4	3	3
	25% Percentile	2.25	4	3	4
	Median	4	5	4	5
	75% Percentile	4	5	4	5
	Maximum	5	5	5	5
	Mean	3.406	4.656	3.313	4.625
	Std. Deviation	1.214	0.4826	0.7071	0.6183
	Std. Error of Mean	0.2147	0.08531	0.1667	0.1457
	Mann Whitney test	**P value: <0.0001**		**P value: 0.0089**	
Q7	Minimum	1	3	3	4
	25% Percentile	2	4	3.75	4
	Median	4	5	4	5
	75% Percentile	4	5	4.25	5
	Maximum	5	5	5	5
	Mean	3.313	4.625	4	4.722
	Std. Deviation	1.23	0.5536	0.686	0.4609
	Std. Error of Mean	0.2174	0.09786	0.1617	0.1086
	Mann Whitney test	**P value: <0.0001**		**P value: 0 0.0021**	
Q8	Minimum	1	1	1	1
	25% Percentile	1	1	1	1
	Median	1	1	1	1

	75% Percentile	1	1.75	1.25	1
	Maximum	2	2	2	2
	Mean	1.188	1.25	1.222	1.167
	Std. Deviation	0.3966	0.4399	0.4278	0.3835
	Std. Error of Mean	0.0701	0.07777	0.1008	0.09039
	Mann Whitney test	**P value: 0.7633**		P value: >0.9999	
**Q9**	Minimum	1	1	1	1
	25% Percentile	3	3	3	3
	Median	3	3	3	3
	75% Percentile	3	3	3	3
	Maximum	3	3	4	3
	Mean	2.719	2.938	2.944	2.889
	Std. Deviation	0.5811	0.3536	0.6391	0.4714
	Std. Error of Mean	0.1027	0.0625	0.1506	0.1111
	Mann Whitney test	P value: 0.0534		P value: 0.6766	
